# The Value of Median Nerve Sonography as a Predictor for Short- and Long-Term Clinical Outcomes in Patients with Carpal Tunnel Syndrome: A Prospective Long-Term Follow-Up Study

**DOI:** 10.1371/journal.pone.0162288

**Published:** 2016-09-23

**Authors:** Alexander Marschall, Anja Ficjian, Martin H. Stradner, Rusmir Husic, Dorothea Zauner, Werner Seel, Nicole E. Simmet, Alexander Klammer, Petra Heizer, Kerstin Brickmann, Judith Gretler, Florentine C. Fürst-Moazedi, Rene Thonhofer, Josef Hermann, Winfried B. Graninger, Stefan Quasthoff, Christian Dejaco

**Affiliations:** 1 Department of Rheumatology and Immunology, Medical University of Graz, Graz, Austria; 2 Department of Neurology, Medical University of Graz, Graz, Austria; University of Palermo, ITALY

## Abstract

**Objectives:**

To investigate the prognostic value of B-mode and Power Doppler (PD) ultrasound of the median nerve for the short- and long-term clinical outcomes of patients with carpal tunnel syndrome (CTS).

**Methods:**

Prospective study of 135 patients with suspected CTS seen 3 times: at baseline, then at short-term (3 months) and long-term (15–36 months) follow-up. At baseline, the cross-sectional area (CSA) of the median nerve was measured with ultrasound at 4 levels on the forearm and wrist. PD signals were graded semi-quantitatively (0–3). Clinical outcomes were evaluated at each visit with the Boston Questionnaire (BQ) and the DASH Questionnaire, as well as visual analogue scales for the patient’s assessment of pain (painVAS) and physician’s global assessment (physVAS). The predictive values of baseline CSA and PD for clinical outcomes were determined with multivariate logistic regression models.

**Results:**

Short-term and long-term follow-up data were available for 111 (82.2%) and 105 (77.8%) patients, respectively. There was a final diagnosis of CTS in 84 patients (125 wrists). Regression analysis revealed that the CSA, measured at the carpal tunnel inlet, predicted short-term clinical improvement according to BQ in CTS patients undergoing carpal tunnel surgery (OR 1.8, p = 0.05), but not in patients treated conservatively. Neither CSA nor PD assessments predicted short-term improvement of painVAS, physVAS or DASH, nor was any of the ultrasound parameters useful for the prediction of long-term clinical outcomes.

**Conclusions:**

Ultrasound assessment of the median nerve at the carpal tunnel inlet may predict short-term clinical improvement in CTS patients undergoing carpal tunnel release, but long-term outcomes are unrelated to ultrasound findings.

## Introduction

Carpal Tunnel Syndrome (CTS) is the most frequent peripheral nerve entrapment syndrome, potentially leading to long-term pain and disability. The socio-economic impact of CTS is immense, given that it is responsible for up to 57% of all costs related to occupational upper-extremity disorders.[1,2] Ultrasound imaging with measurement of the cross-sectional area (CSA) and Power Doppler (PD) signals in the median nerve is a valuable tool for the diagnosis of CTS,[3] but it would be interesting to know whether sonographic findings might also predict the clinical outcome of CTS patients.

A few studies have investigated the prognostic value of CSA in the setting of established CTS undergoing carpal tunnel release (CTR). These studies, however, are limited by small sample size, selection bias and short-term follow-up. Besides, the results of these studies are contradictory: One study by Naranjo et al. including 112 wrists found that patients with a large CSA at baseline had a better outcome after carpal tunnel surgery than those with a smaller CSA.[4] In contrast, Mondelli et al. conducted a study of 67 patients and concluded that a smaller CSA was linked to a higher chance of patient satisfaction after CTR, as measured by the Levine/Boston Questionnaire (BQ).[5] In another study, the baseline CSA was not a significant predictor of the clinical outcome after carpal tunnel release. [6,7]

No data are available on the value of CSA in predicting the outcome of CTS patients with conservative management. Besides, the association between CSA and long-term clinical outcomes as well as the relevance of PD findings for patients’ outcomes remains elusive.

This study aimed to investigate the prognostic value of baseline CSA and PD assessments of median nerve damage for short- and long-term clinical outcomes in a prospective cohort of CTS patients.

## Methods

### Patients

This is a prospective long-term follow-up study of the diagnostic value of median nerve sonography in patients with suspected CTS. Details on patient recruitment and workup have been reported previously.[3] In brief, we recruited patients with suspected CTS between March 2010 and December 2011. The initial study protocol included a baseline and a 3-month follow-up visit with clinical, electrophysiological and sonographic evaluations. Inclusion and exclusion criteria are detailed in S1 Table.[2] The diagnosis of CTS was established by the evaluating neurologist, based on symptoms and nerve conduction studies (NCS) carried out at baseline and at 3 months. The neurologist indicated his confidence in the diagnosis on a scale from 0–100% at each visit. A confidence >90% was accepted as confirmed CTS, whereas CTS was excluded when confidence in the diagnosis was <10%. Wrists deemed as possible CTS (>10%, <90% confidence) at baseline but with definite CTS (>90% confidence) at follow-up or those patients undergoing CTR, were also regarded as confirmed CTS cases. The examining neurologist and the ultrasonographer were blinded to each other’s results.

**Fig 1:**
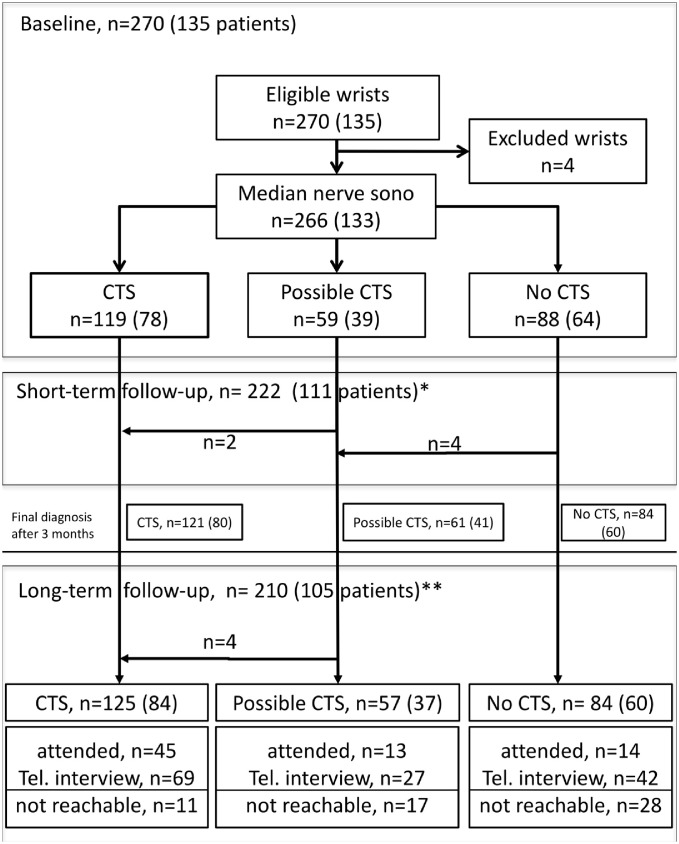
Study flow chart. n, number of wrists; parenthesis: the number of patients in each group (the sum of the numbers in parenthesis might exceed the number of patients under study because patients contributing a wrist to more than 1 group were counted separately); *111 Patients completed short-term follow-up, with 65 of 80 CTS cases included in the regression models; 15 (18.8%) patients had missing or invalid data. **105 Patients completed long-term follow-up, with 74 of 84 CTS cases included in the regression models; 10 (11.9%) patients had missing or invalid data.

After the diagnostic study had been completed, the original study protocol was amended to include assessment of long-term clinical outcomes of the study patients. In January 2013, we phoned all the patients who had undergone the baseline examination and asked them to return for a further clinical follow-up examination. The diagnosis was not re-evaluated at this visit; however, we classified those cases with possible CTS after the 3-month visit and CTR at a later time point as confirmed CTS cases. The visits at baseline and at 3 months as well as the long-term visits included clinical examination and patients’ questionnaires. NCS and ultrasound were performed only at baseline and 3 months. Patients who declined to return or failed to appear for the long-term follow-up visit were contacted again by phone to evaluate their clinical status, pain symptoms and overall quality of life (as detailed below) as well as to address treatments related to CTS and the reason for not returning for follow-up (see Fig 1 for study flow-chart).

Treatment of CTS (including surgery) was not part of the study protocol and was at the discretion of the treating physician. Treatment details were gathered from the patients’ medical history; surgery reports were not available. This study was approved by the institutional review board of the Medical University Graz and written informed consent was obtained by each patient.

### Clinical evaluation

#### Self-administered questionnaires

We used the following scales to evaluate patients’ symptoms: Barriers Questionnaire (BQ,)[8] Disabilities of the Arm Shoulder and Hand (DASH)[9] and a visual analogue scale for the severity of pain (painVAS, range 0-100mm with 0 = best, 100 = worst). The BQ is a two-part self-administered questionnaire for the assessment of the severity of hand symptoms (11 Items) and the functional status of the hand (8 Items). Each item is scored on a 5-point Likert scale (1 = best, 5 = worst) and a mean score for each part is calculated (total score for each part 1–5).[8,10] The DASH comprises 2 parts: the disability/symptom section (30 items, 1–5 Likert scale) and an optional sport/music or work section (4 items, scored 1–5). The assigned values are then transformed into a score ranging from 0 to 100 (0 = best, 100 = worst).[9]

#### Clinical examination

We used the historical-objective scale (Hi-Ob scale) to determine the severity of the disease for each individual wrist.[11] This scale includes dichotomous items concerning history of CTS symptoms (n = 3), clinical findings (n = 3) and a pain item, with a total score ranging from 0 to 5 (0 = best, 5 = worst).[11]

Clinical examination further included evaluation of muscular strength, tropism, sensory function and clinical tests, such as the Phalen's, reverse Phalen's and carpal tunnel compression test with each test scoring “normal” or “abnormal”. In addition, the examiner graded the overall severity of CTS disease using a VAS (physVAS) (range 0-100mm with 0 = best and 100 = worst).

#### Telephone interviews

Telephone assessments of patients who did not come for the long-term follow-up visit were made by 1 investigator and included the following data: PainVAS (scale from 0–100), BQ (1–5), treatment (surgery, splint, pain medication, corticosteroid injection) and reasons for not coming for the examination. The examiner read both the questions and the possible answers to the patients.

#### Evaluation of change of CTS symptoms

Given the absence of established response criteria in CTS, we used the following parameters to evaluate a change of CTS symptoms during follow-up: for the BQ, a 25% improvement of the score compared to baseline as proposed previously.[4] For the DASH and VAS scores, we applied 20% as well as 70% improvements for both scores as relevant outcomes, using the ACR20 and ACR70 response criteria in RA as examples.[12,13] For the VAS, we further specified that a change of less than 10mm was clinically irrelevant given the known intra-rater variability of VAS scores.[14]

### Nerve conduction studies

NCS were performed at baseline and at 3 months by one of two neurologists who were unaware of ultrasound results and used a routine protocol as described previously.[3] In brief, NCS was done on the symptomatic side(s) using commercially available nerve conduction equipment (EMG/NLG/EP-system type Topas; Schwarzer, Munich, Germany). The skin temperature over the dorsum of the hand was kept at 34°C. We determined the antidromic sensory median nerve conduction velocity (NCV, normal values 50 m/s), distal motor latency (DML, 4.2 m) and median motor compound muscle action potential (5 mV).[3]

Since there are no generally acknowledged and standardized grading methods [15–17] and abnormal electrophysiological findings persist after CTR despite clinical improvement [18,19], we were reluctant to include NCS findings as an outcome parameter.

### Ultrasound protocol

One of two rheumatologists experienced in musculoskeletal and nerve sonography (C.De.– 5 years’ experience at the beginning of the study and M.St.– 2 years’ experience) performed ultrasound assessments (baseline and 3-months follow-up visit) as previously described.(3) For the present long-term study, only the baseline results were considered. In brief, we used a Logiq E9 ultrasound device (GE, Milwaukee, WI, USA). B-Mode ultrasound was performed with frequency of 15.0 MHz; PD settings were standardized with a frequency of 11.9 MHz, pulse repetition frequency of 600 Hz and medium persistence. Sampling errors due to differential loads were minimized with a gel pad (thickness 3.3mm; Sonar Aid, Gestlich Pharma, Wolhusen, Switzerland). CSA of the median nerve was determined by tracing a continuous line at the inner hyperechoic rim with electronic calipers. Images were magnified to reduce measurement error.[3]

The CSA of the median nerve was measured between the distal forearm and the carpal tunnel outlet at the following anatomic levels: 1) proximal border of the pronator quadratus muscle (CsP, identified by following the muscle in transverse view to its proximal border), 2) proximal third of the pronator quadratus muscle (CsT, determination of the longitudinal diameter by a longitudinal scan and assessment of the median nerve in transverse view in the proximal third of the muscle), 3) carpal tunnel inlet defined as the proximal margin of the flexor retinaculum (CsR, identification of the flexor retinaculum in transverse view at the level of scaphoid tubercle and pisiform bone and following the retinaculum to its proximal border), and 4) in the carpal tunnel (CsS, transverse scan at the level of the scaphoid tubercle and pisiform bone). See S1 Fig for image examples. Wrist to forearm ratios were calculated, resulting in 4 CSA values: CsS/CsP, CsS/CsT, CsR/CsP and CsR/CsT. PD signals were graded semi-quantitatively at the carpal tunnel inlet (PD-TI) and within the carpal tunnel (PD-TM) from 0–3, with 0 = no PD signal, 1 = one vessel within median nerve, 2 = two or three single or two confluent vessels and 3 = more than three single or more than two confluent vessels. See S2 Fig for image examples. Inter- and intra-observer reliability of ultrasound results was reported previously and was found to be moderate to good.[3]

### Statistical analysis

To investigate the prognostic value of baseline ultrasound findings for the short- and long-term clinical outcome of CTS patients, we focused on patients with confirmed CTS (n = 84).

All statistical analyses were performed using IBM SPSS Statistics (v22.0). Descriptive statistics were used to summarize the data, depicting medians and ranges for continuous non-parametric data, while the mean and standard deviations are presented for parametric data. Distribution of data was tested with the Kolmogorov–Smirnov test (see S2 Table for details). We generated cross tables to analyze proportions and used the chi-square test to determine significant differences. The Mann-Whitney U-test was used to compare independent groups of non-parametric data, whereas paired data were analyzed with the Wilcoxon test. The Friedman test was applied for multiple paired groups.

We created multivariate binary inclusive logistic regression models to investigate the possible association between baseline CSAs, CSA ratios or PD signals with clinical outcomes. In patients with bilateral CTS, we selected the dominant side as indicated by the Hi-Ob scale, choosing the wrist with the higher value. If both wrists had the same score, we used the mean of both sides for all variables. The following dependent variables (binominal: yes/no) were tested at short- and long-term follow-up:

1) ≥25% improvement of the BQ, 2) ≥20% or 3) ≥70% improvement of DASH, 4) ≥20% or 5) ≥70% improvement of physVAS, 6) ≥20% or 7) ≥70% improvement of painVAS. The CSAs, CSA ratios (multiplied by the factor of 10) and median nerve vascularization (dichotomized according to PD-grading 0–1 and 2–3) [3] served as variables of primary interest and the following covariates were included in each logistic regression model: 1)age at inclusion, 2)symptom duration, 3)body mass index, 4)gender. For sensitivity analysis, we applied the regression models in subgroups of patients undergoing surgical or conservative treatments as well as in patients returning for the long-term follow-up visit. Besides, we applied regression models using referral to surgical treatment as the dependent variable. Additional sensitivity analyses were done in the primary models by the exclusion of high leverage cases and cases producing low/high DFBETAs and/or large Cook values.

## Results

### Patients’ characteristics

A total of 135 patients with suspected CTS were included in the study and underwent baseline evaluation. One hundred and eleven (82.2%) patients completed short-term follow-up after 3 months and 105 (77.8%) patients were available for long-term follow-up. Fig 1 provides a study flow chart indicating the number of participants presenting for baseline, short- and long-term follow-up visits.

Details concerning demographic data, clinical characteristics and NCS results of all patients at baseline and both follow-up visits are given in Table 1 and S3 Table. Thirty-nine (46.4% of all CTS patients) patients underwent CTR after the baseline visit (10 patients undergoing CTR before and 29 after first follow-up visit). Forty (47.6%) patients received conservative treatments including splinting and/or NSAIDs therapy. Five (6.0%) patients received no specific treatment.

**Table 1:** Clinical characteristics and nerve conduction studies.

Parameter	Baseline n = 135 patients	Short-term n = 111 patients	Long-term n = 105 patients	p-values
**BQ** ‡	2.1 (0.8–2.7)	1.8 (0.1–2.4)	1 (0–2.8)	0.26; 0.83
**DASH** ‡	30.1 (6.7–59.2)	26.25 (4.1–47.5)	20 (0–45.8)	**0.00; 0.02**
**painVAS** ‡	44 (9–86)	32 (2–58)	19.5 (0–86)	**0.02; 0.02**
**physVAS**‡	45 (0–85)	34 (0–79)	30 (2–77)	0.13; 0.13
**DML**‡*				
** • CTS**	4.7 (3.1–9.3)	4.6 (3.2–8.1)	-	**0.01**
** • No CTS**	3.2 (2.7–4.6)	3.4 (1–4.1)	-	**0.23**
** • Possible CTS**	3.9 (3–5.8)	3.9 (3.2–5.5)	-	**0.00**
**NCV**‡*				
** • CTS**	54.1 (30.5–78.1)	52.8 (38.9–71.9)	-	0.14
** • No CTS**	56.6 (47.6–65)	55.6 (35.5–74.5)	-	0.70
** • Possible CTS**	55 (30.4–66.7)	55.2 (51.4–64.5)	-	0.38
**AMP**‡*				
** • CTS**	7.0 (0.3–12.2)	6.5 (1.6–13.1)	-	0.25
** • No CTS**	9.4 (5.3–14.6)	7.6 (1.6–13.6)	-	**0.01**
** • Possible CTS**	8.6 (4.3–12.9)	9.4 (3.3–10.9)	-	**0.01**

^‡^median (range), Wilcoxon test was used for comparisons of data retrieved at different visits; n, number of patients,

*number of wrists investigated at baseline and at the 3-month follow-up visit were the following: CTS group n = 115 and n = 100, respectively, no CTS n = 43 and n = 32, respectively, possible CTS n = 49 and n = 41, respectively; BQ, Boston Questionnaire; DASH, Disabilities of the Arm, Shoulder and Hand; painVAS, pain Visual analogue Scale; physVAS, Visual Analogue Scale for grading severity of disease (completed by examiner); DML, distal motor latency; NCV, nerve conduction velocity; AMP, amplitudeP-values; first value refers to differences between baseline and short-term follow-up, second value refers to differences between baseline and long-term follow-up

Clinical severity scales (BQ, DASH, painVAS and physVAS) were generally lower at the long-term follow-up visit than at the baseline and short-term follow-up visits (Table 1).

There were no significant differences between CTS patients with complete and incomplete follow-up regarding age at inclusion (57.9 vs 54 years, respectively, p = 0.198), symptom duration (12.8 vs 13.2 months, p = 0.826), BMI (26.9 vs 27.9, p = 0.052) and gender (70% vs 67% females, p = 0.837). Besides, there was no significant difference between patients who came for the long-term follow-up visit and those evaluated by phone concerning gender, symptom duration, BMI, proportion of CTR and baseline clinical scales. Patients not returning for long-term follow-up, however, were younger and more commonly reported an improvement of symptoms compared to baseline. For details, see S4 Table.

### Median nerve sonography

The results of baseline CSA measurement and PD findings of the median nerve at the different anatomical levels are presented in Table 2. As reported previously, patients with CTS had higher values for CsR, CsS, CsR/CsP, CsR/CsT, CsS/CsP and CsS/CsT than patients without CTS and more commonly showed PD signal.[3]

**Table 2:** Cross sectional area (CSA), CSA ratios and PD results at baseline.

	**Ultrasound determined CSA of median nerve at different anatomical levels (mm**^**2**^**)**				**Power Doppler**	
	CsR‡	CsS‡	CsP‡	CsT‡	PD-TI*	PD-TM*
**CTS (n = 119)**	12 (8–25)	11 (7–30)	7 (5–11)	7 (5–12)	51 (40.2)	23 (18.1)
**No CTS (n = 88)**	9 (6–20)	9 (6–21)	7 (5–12)	7 (5–12)	6 (7.1)	2 (2.5)
	**Ultrasound determined CSA ratios of median nerve**					
	CsR/CsP‡	CsR/CsT‡	CsS/CsP‡	CsS/CsT‡		
**CTS (n = 119)**	1.6 (1–4)	1.6 (1–3)	1.5 (0.8–3.3)	1.5 (0.9–3.3)		
**No CTS (n = 88)**	1.3 (1–2.3)	1.3 (1–2.7)	1.3 (0.9–2)	1.2 (0.8–2.1)		

^‡^median (range); n, number of wrists; CsR, cross-sectional area of the median nerve at the carpal tunnel inlet defined as the proximal margin of the flexor retinaculum; CsS, cross-sectional area of the median nerve in the middle of the carpal tunnel, level of the scaphoid tubercle and pisiform bone; CsP, cross-sectional area of the median nerve at the proximal border of the pronator quadratus muscle; CsT, cross-sectional area of the median nerve at the area of the proximal third of the pronator quadratus muscle; PD-TI, Power Doppler signals in the median nerve determined at the carpal tunnel inlet; PD-TM, Power Doppler signals in the median nerve determined in the carpal tunnel

*Dichotomized PD score (0–1 and 2–3): number of wrists with a PD score of 2–3 (%); PD-TI, Power Doppler signals detected in the median nerve at the carpal tunnel inlet; PD-TM, Power Doppler signals detected in the median nerve within the carpal tunnel

### Ultrasound for the prediction of short-term clinical outcomes

One hundred and eleven (82.2%) patients completed the first follow-up visit after 3 months. Out of these, 80 (72.1%) had a confirmed CTS (121 wrists). We observed ≥25% improvement of the BQ in 13 (16.3%) CTS patients. Improvements of ≥20% and ≥70% in the painVAS were observed in 20 (25.0%) and 9 (11.3%) patients, respectively. Sixteen (20.0%) patients and one (1.3%) patient revealed improvements of the physVAS of ≥20% and ≥70%, respectively. In 7 (8.8%) and 3 (3.8%) patients reductions of the DASH of ≥20% and ≥70%, respectively, were found.

Multivariate inclusive regression models were used to explore whether CSAs, CSA ratios and PD signals predicted short-term clinical outcomes.

In the primary analysis of the entire cohort (65/80 patients with complete data included in analysis), a larger CsR predicted a 20% reduction of the physVAS (OR 1.5, p = 0.02), whereas a higher CsS (OR 0.2, p = 0.03) and a higher CsS/CsP ratio (OR 0.6, p = 0.02) were linked with a lower probability for a 20% DASH response. None of the other ultrasound variables was linked with changes of BQ, painVAS, physVAS or DASH (Table 3, results for 70% improvement of painVAS, physVAS or DASH were not significant, and are not shown). In the subgroup analysis of patients undergoing CTR (n = 23), a larger CsR was linked with an improvement of the BQ (OR 1.8; p = 0.05), whereas in patients with conservative treatment this association was not seen. We found no association between PD signals and any of the short-term clinical outcomes in patients with conservative or surgical therapy (see S5 Table for details).

**Table 3:** Prediction of short-term clinical outcomes of CTS patients by baseline ultrasound results.

All CTS Patients(n = 65)**																
	CsR		CsR/Csp*		CsR/CsT*		CsS		CsS/CsP*		CsS/CsT*		PD-TI		PD-TM	
	OR	p	OR	p	OR	p	OR	p	OR	p	OR	p	OR	p	OR	p
**BQ 25%**	1.0	0.80	0.9	0.76	0.9	0.78	0.8	0.29	0.8	0.14	0.8	0.15	1.1	0.89	3.0	0.10
**painVAS 20%**	1.1	0.43	1.0	0.43	1.1	0.33	1.0	0.87	1.0	0.71	1.0	0.58	0.8	0.64	0.6	0.40
**physVAS 20%**	**1.5**	**0.02**	1.3	0.06	1.1	0.21	1.1	0.34	1.1	0.41	1.0	0.62	1.0	0.94	1.5	0.46
**DASH 20%**	0.9	0.44	0.9	0.29	1.0	0.95	**0.2**	**0.03**	**0.6**	**0.02**	0.6	0.07	2.2	0.14	0.2	0.17

OR, odds ratio; p, p-value; BQ 25%, Improvement of at least 25% of the Boston Questionnaire; painVAS 20%, Improvement of at least 20% of the Visual Analogue Scale for the grading of pain symptoms; physVAS20%, Improvement of at least 20% of the Visual Analogue Scale for grading severity of disease (completed by examiner); DASH 20%, Improvement of at least 20% of the Disabilities of the Arm, Shoulder and Hand scale; CsR, cross-sectional area of the median nerve at the carpal tunnel inlet defined as the proximal margin of the flexor retinaculum; CsS, cross-sectional area of the median nerve in the middle of the carpal tunnel, level of the scaphoid tubercle and pisiform bone; CsP, cross-sectional area of the median nerve at the proximal border of the pronator quadratus muscle; CsT, cross-sectional area of the median nerve at the area of the proximal third of the pronator quadratus muscle; PD-TI, Power Doppler signals in the median nerve determined at the carpal tunnel inlet; PD-TM, Power Doppler signals in the median nerve determined in the carpal tunnel;

*ratios multiplied by the factor of 10

**Due to missing data in 15 cases, only 65 of 80 cases were included in the regression models

Non-significant covariates included in each regression model were: (1) age at inclusion, (2) symptom duration, (3) body mass index (BMI), (4) gender. Logistic regression models were conducted as outlined in the Materials & Methods section.

None of the covariates was associated with dependent variables. Exclusion of cases with high leverage, low/high DFBETAs and/or large Cook values did not change these results.

### Ultrasound for the prediction of long-term clinical outcomes

A total of 105 (77.8%) patients were available for long-term follow-up, with 36 (34.3%) patients returning for a long-term follow-up visit after an average of 27.9 months (range 15–36) and 69 (65.7%) patients completing a telephone interview. Seventy-four out of 84 (88%) CTS patients (114 wrists) were evaluated for long-term follow-up [30 patients (45 wrists) pesented for the clinical visit, 44 patients (69 wrists) were evaluated by phone].

Overall, 10 (13.5%) CTS patients showed an improvement of the BQ≥25% compared to baseline. Improvements of ≥20% and ≥70% in the painVAS were seen in 60 (81.1%) and 49 (66.2%) patients, respectively. Thirteen (17.6%) and 9 (12.2%) patients had ≥20% and ≥70% improvements of the physVAS, respectively. We found reductions of the DASH of ≥20% and ≥70% in 9 (12.2%) and 4 (5.4%) patients, respectively.

In the primary analysis including the entire cohort (67/74 patients with complete data), a higher CsS/CsT ratio was associated with a lower probability of a 25% improvement of the BQ, whereas none of the other ultrasound variables predicted a long-term improvement of BQ, painVAS, physVAS or DASH, as detailed in Table 4. No significant association was found in the subgroup analyses of patients undergoing CTR (n = 24) or those with conservative treatment (n = 43), (S6 Table).

**Table 4:** Ultrasound variables for the prediction of long-term clinical outcome.

All CTS Patients (n = 67)**																
	CsR		CsR/CsP*		CsR/CsT*		CsS		CsS/CsP*		CsS/CsT*		PD-TI		PD-TM	
	OR	p	OR	p	OR	p	OR	p	OR	P	OR	p	OR	p	OR	p
**BQ 25%**	1.3	0.11	1.3	0.13	1.2	0.28	0.7	0.17	0.7	0.08	**0.7**	**0.05**	0.7	0.57	3.5	0.10
**painVAS 20%**	1.0	0.81	1.1	0.52	1.1	0.45	0.9	0.30	1.0	0.44	1.0	0.53	0.9	0.95	2.4	0.26
**physVAS 20%**	0.9	0.85	2.1	0.64	1.8	0.66	0.1	0.43	0.0	0.99	0.0	0.99	0.8	0.95	0.4	0.26
**DASH 20%**	0.7	0.15	0.3	0.12	0.1	0.09	0.2	0.35	0.1	0.06	0.0	0.88	0.6	0.43	0.6	0.52

OR, odds ratio; p, p-value; BQ 25%, Improvement of at least 25% of the Boston Questionnaire; painVAS 20%, Improvement of at least 20% of the Visual Analogue Scale for the grading of pain Symptoms; physVAS20%, Improvement of at least 20% of the Visual Analogue Scale for grading severity of disease (completed by examiner); DASH 20%, Improvement of at least 20% of the Disabilities of the Arm, Shoulder and Hand scale; CsR, cross-sectional area of the median nerve at the carpal tunnel inlet defined as the proximal margin of the flexor retinaculum; CsS, cross-sectional area of the median nerve in the middle of the carpal tunnel, level of the scaphoid tubercle and pisiform bone; CsP, cross-sectional area of the median nerve at the proximal border of the pronator quadratus muscle; CsT, cross-sectional area of the median nerve at the area of the proximal third of the pronator quadratus muscle; PD-TI, Power Doppler signals in the median nerve determined at the carpal tunnel inlet; PD-TM, Power Doppler signals in the median nerve determined in the carpal tunnel;

*ratios multiplied by the factor of 10

** Due to missing data in 7 cases, 67 of 74 cases were included in the regression models

Non-significant covariates included in each regression model were: 1) age at inclusion, 2) symptom duration, 3) body mass index (BMI), 4) gender. Logistic regression models were conducted as outlined in the Methods section.

Next, we focused on those patients who presented for all visits (i.e. baseline, short- and long-term follow-up visits). In this group (n = 42), we found that CsR/CsP, CsR/CsT, CsS, CsS/CsP and CsS/CsT predicted long-term improvement of painVAS, physVAS and DASH (OR for significant results ranging from 0.3–0.6; p-values from 0.02–0.05). See S7 Table for details.

Focusing on those patients with CTR who came for all the follow-up visits (n = 12), however, there was no significant association between ultrasound variables and outcomes (S8 Table).

PD signals did not predict long-term clinical outcomes in any of the models. Besides, none of the clinical and demographic covariates included in the models was linked with any of the outcomes.

Next, we calculated a regression model to investigate the possible link between baseline ultrasound parameters and referral to surgical treatment. High baseline CsS/CsP and CsS/CsT ratios (OR = 2.3, p = 0.037 and 2.2, p = 0.054, respectively) as well as age (OR: 1.04–1.05, p-value: 0.011–0.029) were associated with referral to CTR (S9 Table). The exclusion of high leverage cases, cases producing low/high DFBETAs and/or large Cook values did not change the results.

## Discussion

Our data indicate that ultrasound assessment of the median nerve is of limited value for the prediction of short- and long-term clinical outcomes of patients with new CTS. Only in a subgroup of patients undergoing CTR may baseline measurement of CSAs predict an improvement according to the BQ whereas baseline PD findings were not linked with any of the short- or long-term clinical results.

In the subgroup of surgically treated patients, we identified higher CsR as a predictor for improved symptoms after CTR. Our findings are in line with the results of one former trial,[4] but also contradict the findings of 2 other studies.[5,6] It is tempting to speculate that CTR may be more effective in patients with higher median nerve CSA. A high CSA could indicate swelling of the nerve next to the site of compression in the carpal tunnel. Surgical relief of intra-carpal pressure may restore nerve function and reduce symptoms.[1] Other short-term clinical outcomes investigated in our study were not linked with baseline ultrasound findings, and the association between CsR and BQ was not consistent over time. Thus, we have little confidence in the value of ultrasound as a predictor of the outcome of surgical treatment of CTS.

None of the ultrasound variables (i.e. neither CSA nor PD) was consistently linked with short- or long-term clinical outcomes in analyses of the entire cohort (mainly consisting of conservatively managed patients), further casting doubt the value of sonography as a predictor of CTS management. In contrast, the value of ultrasound for diagnosis of CTS is unquestioned, as current studies and a recent meta-analysis indicate.[3,10,20,21]

We have no explanation for the lack of association between baseline ultrasound results and (particularly long-term) clinical outcomes; CSA and vascularization of the median nerve might not sufficiently reflect the nerve pathology/damage in CTS. Reduced mobility and/or flattening of the median nerve, a loss of the fascicular structure and/or thickening of the retinaculum are ultrasound findings in CTS patients that we did no not assess. [20] We cannot say whether any of these signs would better predict the clinical outcome of CTS patients.

The role of clinical factors and baseline NCS values for prediction of short- and long-term clinical outcomes of CTS patients is unclear as well. Some studies, for example, reported a poorer outcome after CTR in patients with upper extremity functional limitations and normal NCS values at baseline.[22,23] Others observed that a shorter distal sensory latency was associated with a higher likelihood of improvement of paresthesia after CTR.[24] Many studies, however, pointed out that neither clinical tests nor NCS parameters could reliably predict a response to treatment.[4,25,26] Median-ulnar sensory latency difference is a very sensitive NCS method for the diagnosis of CTS; its value for outcome prediction, however, is still unclear.[27] Future prospective studies may assess whether a combination of clinical, NCS and ultrasound parameters might better predict the treatment outcome of CTS patients.

The strengths of our study are the prospective design, the long-term follow-up and the inclusion of patients with newly diagnosed CTS in whom the diagnosis was confirmed by clinical and NCS findings. Although we were unable to convince all patients to return for a long-term follow-up examination, we retrieved clinical data by telephone interview from most patients who did not come in for the visit and included these findings into the regression analysis. This prevented us from reporting spurious findings due to selection bias, as might have occurred in previous trials.[6] Associations between ultrasound and clinical items were in fact observed in the subgroup of patients returning for long-term follow-up examinations; however, this effect disappeared when data from telephone interviews were also included in the regression model.

In one sub-analysis, we observed that CsS/CsP (but not the other ultrasound parameters) was linked with the referral to CTR. Although the physician(s) managing CTS patients were unaware of the ultrasound results, we recognize that our study, where treatment decisions were not part of the study protocol, might not have been optimal to answer the question as to whether patients with abnormal ultrasound results are more likely to undergo surgery. Future studies with a prospective randomized design and a pre-specified treatment algorithm would be needed to investigate whether ultrasound is helpful in choosing the best treatment strategy for CTS.[1,28,29]

The most important limitations of our study are the single-center design and the relatively small number of patients who presented for all visits. Although we made every effort to convince patients to come in for the follow-up visits, several declined because their CTS symptoms had improved and they saw no advantage in yet another visit. Telephone interviews to obtain missing clinical data are certainly not ideal, mainly because patients’ answers in the telephone interview could well differ from those they would have given in the setting of a follow-up visit with a written questionnaire.[30]

In conclusion, we found that ultrasound examination of the median nerve at baseline is of limited value for predicting the clinical outcome of CTS patients. In a subgroup of patients undergoing CTR, sonographic determination of cross-sectional area of the median nerve at the carpal tunnel inlet might predict clinical improvement according to the BQ.

## Supporting Information

S1 Fig Image examples for the measurement of the CSA of the median nerve at different anatomical levels in patients with CTS. The CSA of the median nerve was measured between the distal forearm and the carpal tunnel outlet at the following anatomic levels: (A) proximal border of the pronator quadratus muscle, (B) proximal third of the pronator quadratus muscle, (C) carpal tunnel inlet defined as the proximal margin of the flexor retinaculum and (D) in the carpal tunnel.
(TIF)

S2 Fig Examples for semi-quantitative scoring (0–3) of power Doppler (PD) signals. PD signals within the median nerve were semi-quantitatively graded from 0 to 3 as outlined in Materials and Methods. Examples show transverse scans of the median nerve at the carpal tunnel inlet with PD scores ranging from zero (PD = 0) to three (PD = 3).
(TIF)

S1 Table Inclusion and exclusion criteria. (DOCX)

S2 Table Kolmogorov-Smirnov Test results. (DOCX)

S3 Table Demographic and clinical characteristics of CTS Patients at baseline. (DOCX)

S4 Table Clinical characteristics of patients present at long-term follow-up visit vs patients evaluated by phone. (DOCX)

S5 Table Prediction of short-term clinical outcomes of CTS patients by baseline ultrasound results. (DOCX)

S6 Table Prediction of long-term clinical outcomes of CTS patients by baseline ultrasound results. (DOCX)

S7 Table Prediction of long-term clinical outcomes of CTS patients who presented for all three follow-up visits by baseline ultrasound results. (DOCX)

S8 Table Logistic regression models for long-term outcome in CTS Patients who underwent CTR and all three follow-up visits. (DOCX)

S9 Table Logistic regression models predicting CTR by baseline ultrasound results. (DOCX)
