# Supplementary figures and images for: The Value of Median Nerve Sonography as a Predictor for Short- and Long-Term Clinical Outcomes in Patients with Carpal Tunnel Syndrome: A Prospective Long-Term Follow-Up Study
Source: PLoS One. 2016 Sep 23;11(9):e0162288. doi: 10.1371/journal.pone.0162288 (PMC5035047; doi:10.1371/journal.pone.0162288)

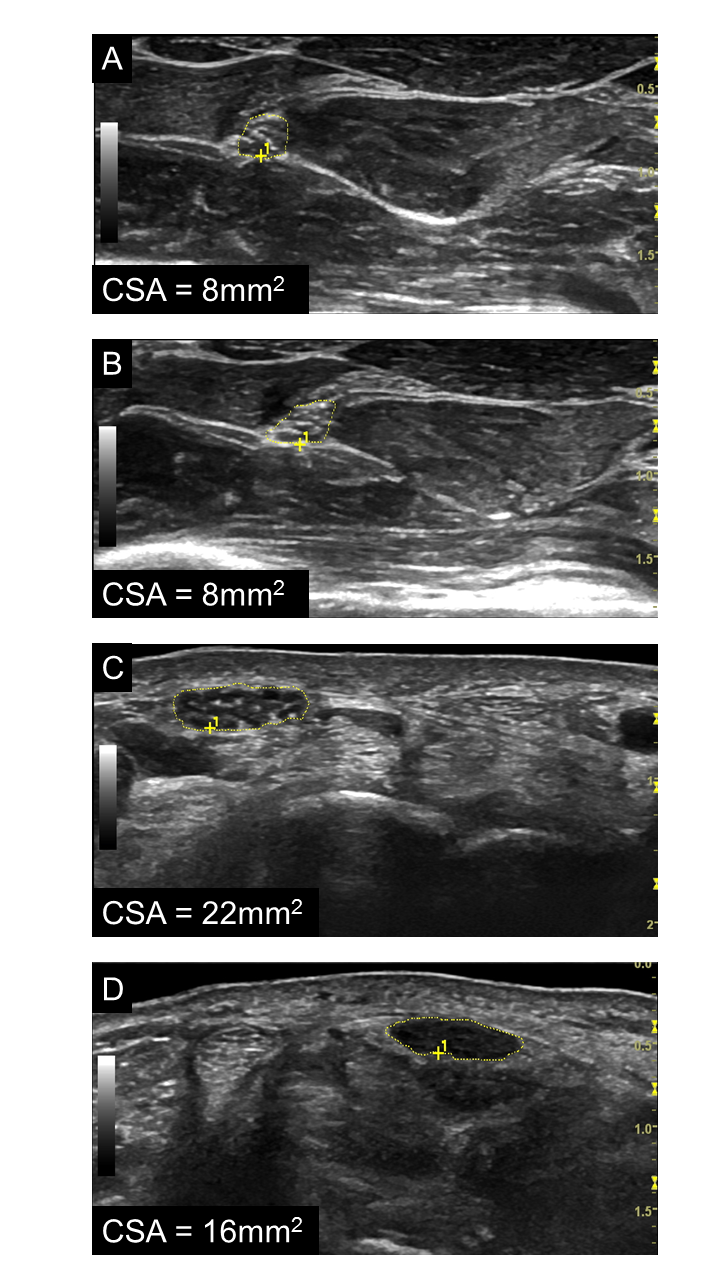

Supplement: S1 Fig — The CSA of the median nerve was measured between the distal forearm and the carpal tunnel outlet at the following anatomic levels: (A) proximal border of the pronator quadratus muscle, (B) proximal third of the pronator quadratus muscle, (C) carpal tunnel inlet defined as the proximal margin of the flexor retinaculum and (D) in the carpal tunnel. (TIF) [file pone.0162288.s001.tif]

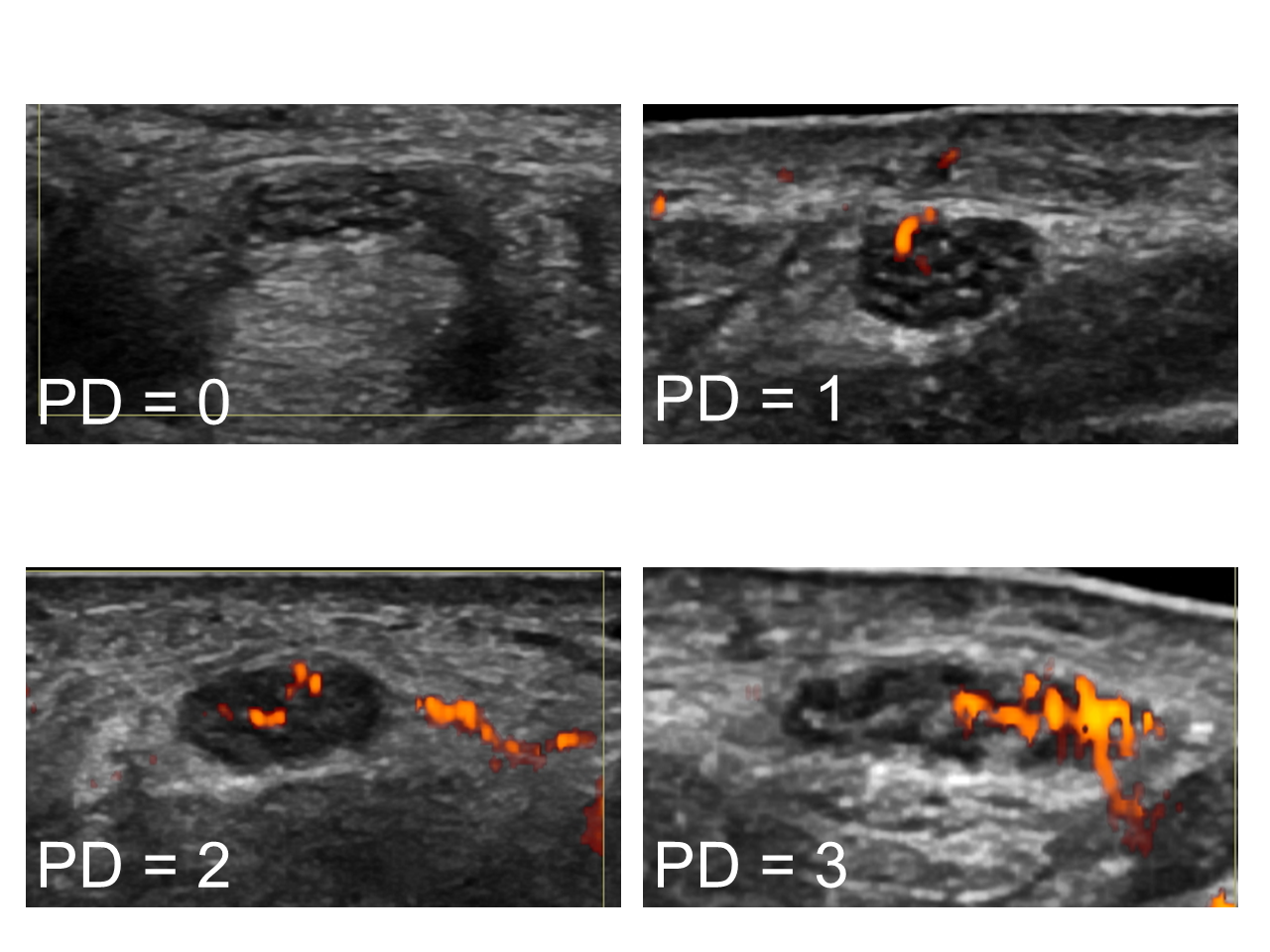

Supplement: S2 Fig — PD signals within the median nerve were semi-quantitatively graded from 0 to 3 as outlined in Materials and Methods. Examples show transverse scans of the median nerve at the carpal tunnel inlet with PD scores ranging from zero (PD = 0) to three (PD = 3). (TIF) [file pone.0162288.s002.tif]
