# Supplementary material for: The Value of Median Nerve Sonography as a Predictor for Short- and Long-Term Clinical Outcomes in Patients with Carpal Tunnel Syndrome: A Prospective Long-Term Follow-Up Study
Source: PLoS One. 2016 Sep 23;11(9):e0162288. doi: 10.1371/journal.pone.0162288 (PMC5035047; doi:10.1371/journal.pone.0162288)
Supplement: S1 Table — (DOCX) [file pone.0162288.s003.docx]

S1 Table: Inclusion and exclusion criteria

| Inclusion criteria† | Exclusion Criteria‡ |
| --- | --- |
| paresthesia, pain and/or sensory deficits in the hand in a median nerve distribution | previously diagnosed CTS |
| nocturnal/ early morning worsening of paresthesia with disturbed sleep | former wrist surgery |
| paresthesia relieved by hand movement or shaking | recent wrist fracture |
| pain and/or paresthesia in a median nerve distribution provoked by monotonous exercises | known inflammatory rheumatic disease |
| weakness of fingers supplied by median nerve | pregnancy |
|  | known polyneuropathy |
|  | contraindications for electrophysiological testing |

†Patients were included who presented at least one of the symptoms at one or both wrists; ‡Patients who met at least one of the exclusion criteria were excluded
