# Supplementary material for: The Value of Median Nerve Sonography as a Predictor for Short- and Long-Term Clinical Outcomes in Patients with Carpal Tunnel Syndrome: A Prospective Long-Term Follow-Up Study
Source: PLoS One. 2016 Sep 23;11(9):e0162288. doi: 10.1371/journal.pone.0162288 (PMC5035047; doi:10.1371/journal.pone.0162288)
Supplement: S2 Table — (DOCX) [file pone.0162288.s004.docx]

S2 Table: Kolmogorov-Smirnov test results

| **Parameter** | **p-value** | **Distribution of data** |
| --- | --- | --- |
| **median nerve values (n=266)** |  |  |
| **CsR** | <0.01 | not normally |
| **CsS** | <0.01 | not normally |
| **CsP** | <0.01 | not normally |
| **CsT** | <0.01 | not normally |
| **PD-TI** | <0.01 | not normally |
| **PD-TM** | <0.01 | not normally |
| **median nerve ratios (n=266)** |  |  |
| **CsR/CsP** | 0.20 | normally |
| **CsR/CsT** | 0.08 | not normally |
| **CsS/CsP** | 0.02 | not normally |
| **CsS/CsT** | 0.20 | normally |
| **nerve conduction studies (n=207)** |  |  |
| **DML** | <0.01 | not normally |
| **NCV** | 0.02 | not normally |
| **AMP** | 0.20 | normally |
| **clinical parameters** |  |  |
| **DASH** | <0.01 | not normally |
| **physVAS** | 0.06 | not normally |
| **painVAS** | 0.09 | not normally |
| **BQ** | <0.01 | not normally |

CsR, cross-sectional area of the median nerve at the carpal tunnel inlet defined as the proximal margin of the flexor retinaculum; CsS, cross-sectional area of the median nerve in the middle of the carpal canal, level of the scaphoid tubercle and pisiform bone; CsP, cross-sectional area of the median nerve at the proximal border of the pronator quadratus muscle; CsT, cross-sectional area of the median nerve at the area of the proximal third of the pronator quadratus muscle; PD-TI, Power Doppler signals in the median nerve determined at the carpal tunnel inlet; PD-TM, Power Doppler signals in the median nerve determined in the carpal canal; DML, distal motor latency; NCV, nerve conduction velocity; AMP, amplitude

n=number of wrists included in the analysis; BQ, Boston Questionnaire; DASH, Disabilities of the Arm, Shoulder and Hand; painVAS, pain Visual Analogue Scale; physVAS, Visual Analogue Scale for grading severity of disease (completed by examiner)
