# Supplementary material for: The Value of Median Nerve Sonography as a Predictor for Short- and Long-Term Clinical Outcomes in Patients with Carpal Tunnel Syndrome: A Prospective Long-Term Follow-Up Study
Source: PLoS One. 2016 Sep 23;11(9):e0162288. doi: 10.1371/journal.pone.0162288 (PMC5035047; doi:10.1371/journal.pone.0162288)
Supplement: S3 Table — (DOCX) [file pone.0162288.s005.docx]

S3 Table: Demographic and clinical characteristics of CTS patients at baseline

| parameter | baseline result |
| --- | --- |
| age at inclusion [years] † | 51.9 (±14.5) |
| female, n (%) | 99 (73.3) |
| body mass index [kg/m­^2^]† | 26.8 (±4.3) |
| symptom duration (months) ‡ | 12 (1-362) |
| ESR [mm/1st hour] † | 11.2 (±10) |
| CRP [mg/l] ‡ | 1.4 (0.6 – 26.2) |
| employment, n (%) |  |
| - blue collar | 51 (38.3) |
| - white collar | 49 (36.8) |
| - housewife/domestic | 23 (17.3) |
| - retired | 4 (3) |
| - other | 6 (4.5) |
| manual hobbies, n (%) | 79 (58.5) |

‡median (range), Wilcoxon test was used for comparisons of data retrieved at different visits; †mean (standard deviation); n, number of patients; ESR, erythrocyte sedimentation rate (normal values 1-10 mm/1st hour); CRP, C-reactive protein (normal values 0-5 mg/L)
