# Supplementary material for: The Value of Median Nerve Sonography as a Predictor for Short- and Long-Term Clinical Outcomes in Patients with Carpal Tunnel Syndrome: A Prospective Long-Term Follow-Up Study
Source: PLoS One. 2016 Sep 23;11(9):e0162288. doi: 10.1371/journal.pone.0162288 (PMC5035047; doi:10.1371/journal.pone.0162288)
Supplement: S4 Table — (DOCX) [file pone.0162288.s006.docx]

S4 Table: Clinical characteristics of patients presenting for long-term follow-up visit vs patients evaluated by phone

|  | long-term follow-up visit, n= 36 | long-term follow-up phone, n= 69 | p-Value |
| --- | --- | --- | --- |
| age at inclusion [years] † | 57.53 (±8.96) | 49.90 (±15.75) | 0.005 |
| symptom duration (months) ‡ | 13.15 (2.13 – 122.80) | 12.33 (1.20 – 164.47) | 0.164 |
| body mass index [kg/m­^2^]† | 26.72 (±4.31) | 27.42 (±4.49) | 0.199 |
| females, n (%) | 27 (75) | 54 (78.3) | 0,807 |
| DASH (baseline)† | 29.19 (±21.56) | 30.81 (±17.25) | 0.635 |
| painVAS (baseline) † | 47.63 (±20.66) | 49.03 (±21.36) | 0.912 |
| surgery, n (%) | 16 (44.4) | 23 (32.8) | 0.282 |
| painVAS 20%, n (%) | 15 (41.7) | 42 (60.9) | 0.067 |
| painVAS 70%, n (%) | 11 (30.6) | 37 (53.6) | 0.038 |

‡median (range); †mean (standard deviation); n, number of patients;

DASH, disabilities of the arm, shoulder and hand questionnaire; VAS, visual analogue scale
