# Supplementary material for: The Value of Median Nerve Sonography as a Predictor for Short- and Long-Term Clinical Outcomes in Patients with Carpal Tunnel Syndrome: A Prospective Long-Term Follow-Up Study
Source: PLoS One. 2016 Sep 23;11(9):e0162288. doi: 10.1371/journal.pone.0162288 (PMC5035047; doi:10.1371/journal.pone.0162288)
Supplement: S6 Table — (DOCX) [file pone.0162288.s008.docx]

**S6 Table**: Prediction of long-term clinical outcomes of CTS patients by baseline ultrasound results

| **conservative treatment (n=43)** | | | | | | | | | | | | | | | | |
| --- | --- | --- | --- | --- | --- | --- | --- | --- | --- | --- | --- | --- | --- | --- | --- | --- |
|  | CsR | | CsR/CsP* | | CsR/CsT* | | CsS | | CsS/CsP* | | CsS/CsT* | | PD-TI | | PD-TM | |
|  | OR | p | OR | p | OR | p | OR | p | OR | p | OR | p | OR | p | OR | p |
| **BQ 25%** | 1.3 | 0.19 | 0.7 | 0.1 | 2.2 | 0.4 | 1 | 0.85 | 1.1 | 0.99 | 0 | 0.99 | 2.2 | 0.98 | 0.2 | 0.61 |
| **painVAS 20%** | 0.9 | 0.85 | 1.1 | 0.64 | 2 | 0.18 | 0.1 | 0.43 | 1.1 | 0.99 | 1 | 0.98 | 0.7 | 0.85 | 0.9 | 0.93 |
| **physVAS 20%** | 0 | 0.99 | 3.4 | 0.99 | 3.1 | 0.99 | 0.5 | 0.34 | 3.9 | 0.99 | 5.5 | 0.2 | 1 | 0.99 | 1.2 | 0.98 |
| **DASH 20%** | 1.2 | 0.66 | 5.7 | 0.99 | 1.1 | 0.29 | 1.6 | 0.61 | 3.7 | 0.99 | 0.1 | 0.71 | 0.7 | 0.23 | 1.5 | 0.72 |
| **CRT (n=24)** | | | | | | | | | | | | | | | | |
|  | CsR | | CsR/Csp* | | CsR/CsT* | | CsS | | CsS/CsP* | | CsS/CsT* | | PD-TI | | PD-TM | |
|  | OR | p | OR | p | OR | p | OR | p | OR | p | OR | P | OR | p | OR | p |
| **BQ 25%** | 1.9 | 0.09 | 1.6 | 0.13 | 1.4 | 0.14 | 0.3 | 0.17 | 0.6 | 0.32 | 1 | 0.61 | 0.2 | 0.25 | 1.2 | 0.91 |
| **painVAS 20%** | 1 | 0.81 | 1.1 | 0.52 | 1.1 | 0.45 | 0.9 | 0.3 | 1 | 0.44 | 1 | 0.53 | 0.8 | 0.1 | 0.6 | 0.37 |
| **physVAS 20%** | 0.9 | 0.36 | 0.7 | 0.41 | 0.2 | 0.29 | 0.7 | 0.11 | 0.1 | 0.08 | 0.1 | 0.06 | 0.7 | 0.2 | 0.9 | 0.99 |
| **DASH 20%** | 0.7 | 0.15 | 0.1 | 0.12 | 0.1 | 0.09 | 0.2 | 0.35 | 0 | 0.06 | 0 | 0.08 | 0.8 | 0.67 | 1.2 | 0.62 |

OR= odds ratio; p, p-value; CTR, carpal tunnel release; BQ 25%, improvement of at least 25% of the Boston Questionnaire; painVAS 20%, improvement of at least 20% of the Visual Analogue Scale for the grading of pain symptoms; physVAS20%, improvement of at least 20% of the Visual Analogue Scale for grading severity of disease (completed by examiner); DASH 20%, improvement of at least 20% of the Disabilities of the Arm, Shoulder and Hand scale; CsR, cross-sectional area of the median nerve at the carpal tunnel inlet defined as the proximal margin of the flexor retinaculum; CsS, cross-sectional area of the median nerve in the middle of the carpal canal, level of the scaphoid tubercle and pisiform bone; CsP, cross-sectional area of the median nerve at the proximal border of the pronator quadratus muscle; CsT, cross-sectional area of the median nerve at the area of the proximal third of the pronator quadratus muscle; PD-TI, Power Doppler signals in the median nerve determined at the carpal tunnel inlet; PD-TM, Power Doppler signals in the median nerve determined in the carpal canal; *ratios multiplied by a factor of 10

Non-significant covariates included in each regression model were: 1) age at inclusion, 2) symptom duration, 3) body mass index (BMI), 4) gender. Logistic regression models were conducted as outlined in the methods section.
