# Supplementary material for: The Value of Median Nerve Sonography as a Predictor for Short- and Long-Term Clinical Outcomes in Patients with Carpal Tunnel Syndrome: A Prospective Long-Term Follow-Up Study
Source: PLoS One. 2016 Sep 23;11(9):e0162288. doi: 10.1371/journal.pone.0162288 (PMC5035047; doi:10.1371/journal.pone.0162288)
Supplement: S7 Table — (DOCX) [file pone.0162288.s009.docx]

S7 Table: Prediction of long-term clinical outcomes of CTS patients presenting at all three follow-up visits by baseline ultrasound results

| **CTS patients undergoing all three follow-up visits (n=42)** | | | | | | | | | | | | | |  |
| --- | --- | --- | --- | --- | --- | --- | --- | --- | --- | --- | --- | --- | --- | --- |
|  | CsR | | CsR/CsP* | | CsR/CsT* | | CsS | | CsS/CsP* | | CsS/CsT* | | |  |
|  | OR | p | OR | p | OR | p | OR | p | OR | p | OR | p | | |
| **BQ 25%** | 1.4 | 0.08 | 1.3 | 0.08 | 1.1 | 0.06 | 0.7 | 0.28 | 0.9 | 0.18 | 0.2 | 0.18 |  |  |
| **painVAS 20%** | 1.0 | 0.63 | 1.2 | 0.36 | 1.1 | 0.32 | **0.3** | **0.04** | **0.4** | **0.04** | **0.4** | **0.05** |  |  |
| **physVAS 20%** | 0.9 | 0.39 | 0.8 | 0.13 | 0.8 | 0.69 | 0.7 | 0.07 | **0.6** | **0.03** | **0.6** | **0.03** |  |  |
| **DASH 20%** | 0.7 | 0.11 | **0.6** | **0.04** | **0.6** | **0.04** | **0.4** | **0.04** | **0.5** | **0.02** | **0.3** | **0.03** |  |  |
| **painVAS 70%** | 0.9 | 0.55 | 1.0 | 0.87 | 1.0 | 0.91 | **0.3** | **0.03** | **0.4** | **0.04** | 0.4 | 0.07 |  |  |
| **physVAS 70%** | 1.0 | 0.75 | 0.8 | 0.21 | 0.9 | 0.47 | 0.7 | 0.18 | 0.7 | 0.07 | 0.8 | 0.12 |  |  |
| **DASH 70%** | 0.8 | 0.39 | 0.6 | 0.12 | 0.7 | 0.14 | 0.6 | 0.11 | 0.5 | 0.08 | 0.5 | 0.08 |  |  |

OR= odds ratio; p, p-value;

BQ 25%, improvement of at least 25% of the Boston Questionnaire; painVAS 20%/70%, improvement of at least 20%/70% of the visual analogue scale for the grading of pain symptoms; physVAS20%/70%, improvement of at least 20%/70% of the visual analogue scale for grading severity of disease (completed by examiner); DASH 20%/70%, improvement of at least 20%/70% of the Disabilities of the Arm, Shoulder and Hand scale; CsR, cross-sectional area of the median nerve at the carpal tunnel inlet defined as the proximal margin of the flexor retinaculum; CsS, cross-sectional area of the median nerve in the middle of the carpal canal, level of the scaphoid tubercle and pisiform bone; CsP, cross-sectional area of the median nerve at the proximal border of the pronator quadratus muscle; CsT, cross-sectional area of the median nerve at the area of the proximal third of the pronator quadratus muscle; PD-TI, Power Doppler signals in the median nerve determined at the carpal tunnel inlet; PD-TM, Power Doppler signals in the median nerve determined in the carpal canal; *ratios multiplied by the factor of 10
