# Supplementary material for: The Value of Median Nerve Sonography as a Predictor for Short- and Long-Term Clinical Outcomes in Patients with Carpal Tunnel Syndrome: A Prospective Long-Term Follow-Up Study
Source: PLoS One. 2016 Sep 23;11(9):e0162288. doi: 10.1371/journal.pone.0162288 (PMC5035047; doi:10.1371/journal.pone.0162288)
Supplement: S8 Table — (DOCX) [file pone.0162288.s010.docx]

**S8 Table**: Logistic regression models for long-term outcome in CTS patients who underwent CTR and presented for all three follow-up visits

| CTS patients undergoing CTR and all three follow-up visits (n=12) | | | | | | | | | | | | |
| --- | --- | --- | --- | --- | --- | --- | --- | --- | --- | --- | --- | --- |
|  | CsR | | CsR/CsP* | | CsR/CsT* | | CsS | | CsS/CsP* | | CsS/CsT* | |
|  | OR | p | OR | p | OR | p | OR | p | OR | p | OR | p |
| painVAS 20% | 1.5 | 0.34 | 1.0 | 0.89 | 1.1 | 0.69 | 0.0 | 0.99 | -† | | -† | |
| DASH 20% | 0.7 | 0.54 | 0.0 | 1.00 | 0.0 | 0.99 | 0.0 | 1.00 | -† | | -† | |
| physVAS 20% | 0.8 | 0.63 | 0.0 | 0.99 | 0.5 | 0.16 | 0.8 | 0.74 | 0.7 | 0.29 | 0.7 | 0.29 |
| painVAS 70% | 0.9 | 0.89 | 0.0 | 1.00 | 0.6 | 0.29 | 0.0 | 1.00 | -† | | -† | |
| DASH 70% | 0.7 | 0.54 | 0.0 | 1.00 | 0.0 | 1.00 | 0.0 | 1.00 | -† | | -† | |
| physVAS 70% | 0.8 | 0.63 | 0.0 | 0.99 | 0.5 | 0.16 | 0.8 | 0.74 | 0.7 | 0.29 | 0.7 | 0.29 |

OR, odds ratio; p, p-value; 20%, painVAS 20%/70%, improvement of at least 20%/70% of the visual analogue scale for the grading of pain symptoms; physVAS20%/70%, improvement of at least 20%/70% of the visual analogue scale for grading severity of disease (completed by examiner); DASH 20%/70%, improvement of at least 20%/70% of the Disabilities of the Arm, Shoulder and Hand scale; CsR, cross-sectional area of the median nerve at the carpal tunnel inlet defined as the margin of the flexor retinaculum; CsS, cross-sectional area of the median nerve in the middle of the carpal canal, level of the scaphoid tubercle and pisiform bone; CsP, cross-sectional area of the median nerve at the proximal border of the pronator quadratus muscle; CsT, cross-sectional area of the median nerve at the area of the proximal third of the pronator quadratus muscle.

*ratios multiplied by the factor of 10; † insufficient data to conduct regression analysis.
