# Supplementary material for: The Value of Median Nerve Sonography as a Predictor for Short- and Long-Term Clinical Outcomes in Patients with Carpal Tunnel Syndrome: A Prospective Long-Term Follow-Up Study
Source: PLoS One. 2016 Sep 23;11(9):e0162288. doi: 10.1371/journal.pone.0162288 (PMC5035047; doi:10.1371/journal.pone.0162288)
Supplement: S9 Table — (DOCX) [file pone.0162288.s011.docx]

S9 Table: Logistic regression models predicting CTR by baseline ultrasound results

| Prediction CTS Patients, (n=133) | | | | | | | | | | | | | | | | | | | | | | | | | | | | |  |
| --- | --- | --- | --- | --- | --- | --- | --- | --- | --- | --- | --- | --- | --- | --- | --- | --- | --- | --- | --- | --- | --- | --- | --- | --- | --- | --- | --- | --- | --- |
|  | CsR | | CsR/CsP* | | | CsR/CsT* | | | CsS | | | | CsS/CsP* | | | | CsS/CsT* | | | | PD-TI | | | PD-TM | | | | |  |
|  | OR | p | | OR | p | | OR | p | | OR | | p | | OR | | p | | OR | | p | | OR | p | | OR | | p | | |
| **CTR** | 1.0 | 0.93 | | 1.9 | 0.2 | | 1.8 | 0.20 | | 1.1 | 0.26 | | | **2.3** | **0.04** | | | **2.1** | **0.05** | | | 1.1 | 0.77 | | 1.2 | 0.72 | | | |
| **incl. age** | **1.0** | **0.03** | | **1.0** | **0.02** | | **1.0** | **0.02** | | **1.0** | **0.02** | | | **1.1** | **0.01** | | | **1.0** | **0.01** | | | **1.0** | **0.03** | | **1.0** | | | **0.02** | |

OR, odds ratio; p, p-value;

CsR, cross-sectional area of the median nerve at the carpal tunnel inlet defined as the margin of the flexor retinaculum; CsS, cross-sectional area of the median nerve in the middle of the carpal canal, level of the scaphoid tubercle and pisiform bone; CsP, cross-sectional area of the median nerve at the proximal border of the pronator quadratus muscle; CsT, cross-sectional area of the median nerve at the area of the proximal third of the pronator quadratus muscle

PD-TI; Power Doppler at the tunnel inlet defined as the margin of the flexor retinaculum; PD-TM, Power Doppler in the middle of the carpal canal;

*ratios multiplied by a factor of 10; incl. age, refers to the model including age as a covariate in the model
